# Optimization of Injection Molding Parameters for HDPE/TiO_2_ Nanocomposites Fabrication with Multiple Performance Characteristics Using the Taguchi Method and Grey Relational Analysis

**DOI:** 10.3390/ma9080710

**Published:** 2016-08-22

**Authors:** Hifsa Pervez, Mohammad S. Mozumder, Abdel-Hamid I. Mourad

**Affiliations:** 1 Chemical & Petroleum Engineering Department, College of Engineering, UAE University, Al Ain 15551, UAE; hifsa.p@uaeu.ac.ae; 2 Mechanical Engineering Department, College of Engineering, UAE University, Al Ain 15551, UAE; ahmourad@uaeu.ac.ae

**Keywords:** HDPE/TiO_2_ nanocomposites, injection molding parameters, optimization, Taguchi method, grey relational analysis

## Abstract

The current study presents an investigation on the optimization of injection molding parameters of HDPE/TiO_2_ nanocomposites using grey relational analysis with the Taguchi method. Four control factors, including filler concentration (i.e., TiO_2_), barrel temperature, residence time and holding time, were chosen at three different levels of each. Mechanical properties, such as yield strength, Young’s modulus and elongation, were selected as the performance targets. Nine experimental runs were carried out based on the Taguchi L_9_ orthogonal array, and the data were processed according to the grey relational steps. The optimal process parameters were found based on the average responses of the grey relational grades, and the ideal operating conditions were found to be a filler concentration of 5 wt % TiO_2_, a barrel temperature of 225 °C, a residence time of 30 min and a holding time of 20 s. Moreover, analysis of variance (ANOVA) has also been applied to identify the most significant factor, and the percentage of TiO_2_ nanoparticles was found to have the most significant effect on the properties of the HDPE/TiO_2_ nanocomposites fabricated through the injection molding process.

## 1. Introduction

In recent years, polymers have rapidly evolved from basic manufacturing materials to more advanced precursors to sophisticated structures and composites consisting of multi-layered polymeric blends, composites and fibers [1]. In biomedical engineering, for instance, polymeric composites have been widely studied for scaffold design based on a combination of different polymers enriched with various organic and inorganic fillers. Among potential polymers, high density polyethylene (HDPE) is a thermoplastic polymer used in biomedical applications, especially in bone tissue engineering and reconstructive dentistry [2,3,4]. Titanium dioxide, on the other hand, is an important bioactive filler that has been studied extensively by researchers and is known to exhibit low cytotoxicity and negligible immune response [5,6,7]; and recent studies [8,9] also show that TiO_2_ thin films are effective in inactivating bacteria, such *Escherichia coli* (*E. coli*) under simulated sunlight. The physiological activity of titania- and polyethylene-based implants has been studied through various in vivo studies [5,10,11,12,13] and has been shown to improve biocompatibility and to elicit low immune response and no damage to the organs surrounding the implant. Moreover, to reduce unwanted protein adsorption on the surface of the nanocomposites, further hydrophilic coatings and pre-treatments may be adopted to tailor the surface properties [14].

The latest statistics [15] reveals that approximately 300 million metric tons of plastic are being manufactured per annum, of which 11% is in the form of HDPE [16]; given this boom in the polymer industry, several manufacturing processes, including injection molding, extrusion, melt blending and sintering, are being pursued by the industries [1,16,17,18]. Among them, injection molding has successfully been employed in the fabrication of a variety of polymeric composites with different metallic and ceramic fillers [11,19,20,21,22]. It is an essential manufacturing process for polymers, since the resulting products are known for their high dimensional accuracy and precision, the least number of manufacturing cycles and low cost [23,24,25]. However, the literature [26,27,28,29,30] suggests that injection molding operating conditions have a considerable effect on the quality of the end products and play a crucial role in the mechanical properties of the composites. Consequently, optimizing the injection molding process parameters is imperative to the polymer industry [23].

Optimization of a particular process can be seen as either an improvement to an existing set of procedures or the development of further designs to maximize the feasibility of the employed process since the goal of modern industries is to balance low cost with high quality products and minimal waste [31,32]. In injection molding, as mentioned before, process optimization is a well-characterized issue, and considerable work has been done to optimize the process using single [33,34,35] and multiple criteria [31,36,37]. While single attribute decision making has been employed to improve processing conditions, recent sophisticated fabrication processes require a multiple attribute decision making (MADM) approach that takes different criteria into consideration to select an optimum set of parameters [38]. Some common methodologies for solving MADM problems include simple additive weighting (SAW), the analytical hierarchy process (AHP), the technique for order of preference by similarity to ideal solution (TOPSIS), etc. [39,40,41]; nevertheless, most methods require large amounts of data and fail to provide reliable results if the data are uncertain or if there are interactions present between the different factors [38,39].

Grey relational analysis, a part of the grey system theory first developed by J.L. Deng [42], is a more recent and comprehensive approach for solving MADM problems; it is very useful when either the data available are incomplete or uncertain or the relationship between the various attributes is complex, since it applies a method of approximation using a grey relational grade to consider all performance values of an MADM into a single value [32,38]. The analysis follows a certain sequence of procedures that ultimately result in the determination of the grey relational grade, which is translated as the best choice for a particular alternative [38].

Complementing the grey relational analysis is the Taguchi method that attempts to calculate the signal-to-noise (S/N) ratios of the different attributes for the optimization of the process by improving the desired characteristics, while reducing the effect of noise and defects at the same time [16,43]; moreover, Taguchi orthogonal arrays [44] provide a mechanism for the design of experiments based on the number of factors and control levels. More specifically, an orthogonal array denoted by $OA_{N}\left(s^{m}\right)$ is an N×m matrix with ‘*N*’ rows and ‘m’ columns, with ‘s’ being the elements of the system [45]. The unique characteristic of such an array is that any particular ordered pair of elements appears the same number of times in all of the columns. The most commonly-used arrays are L_8_, L_9_, L_16_ and, sometimes, L_32_ [45]. Grey relational analysis combined with Taguchi arrays has been used to optimize many injection molded parameters, the results of which varied by different starting materials [16,23,25,31,46,47,48,49].

Moreover, analysis of variance (ANOVA) is a common statistical method used in optimization studies to identify the control factors that have the highest contribution in the properties of the materials, similar to regression in that it investigates and models the relationship between a response variable and one or more independent variables by analyzing the sample means. In a study by Yang [24], Young’s modulus, ultimate stress and strain variation were the performance attributes, and it was concluded that melt temperature had the most prominent effect on the mechanical properties of the polycarbonate composites reinforced with glass fiber and PTFE. Moreover, Muhammad et al. [45] employed the grey relational analysis along with an L_27_ Taguchi orthogonal array to optimize injection molding parameters to produce Ti-6Al-4V composites. At 90% confidence level, from ANOVA, the injection temperature was found to have the most significant effect on the performance characteristics, such as strength and density [45]. Furthermore, in optimizing the turning operation parameters for the SKD 11 alloy, Tzeng et al. [32] concluded that the depth of cut was the most important factor affecting the roughness and roundness of the material. On the other hand, Jamaludin et al. [46] and Ibrahim et al. [48] found that mold temperature and holding time were the most significant parameters, respectively. Besides injection molding, optimization studies have also been conducted for other manufacturing processes, such as turning and spinning [32,43,50,51,52], and also applied to time variate series [52].

This study aims to optimize the injection molding parameters to prepare optimum nano-TiO_2_-enriched HDPE composites for their potential in biomedical applications. A Taguchi L_9_ (3^4^) orthogonal array has been used in the design of experiments with four controlling factors, namely the concentration of TiO_2_ nanoparticles, the barrel temperature, the residence time and holding time; each of these factors has been identified at three distinct levels. Nine different experimental runs were performed to obtain data for the multiple response characteristics of tensile testing, which include yield strength, modulus of elasticity and elongation at break. Grey relational analysis was chosen to optimize the operating conditions for the fabrication of optimum quality HDPE/TiO_2_ nanocomposites. Lastly, ANOVA was also used to determine the most significant factors for the injection molding technique when a number of mechanical properties were assessed quantitatively.

## 2. Experimental Method

### 2.1. Materials

Nano-TiO_2_-enriched high density polyethylene (HDPE) nanocomposites are the material under the current study. The raw materials, HDPE, were purchased from Sigma Aldrich (Dubai, UAE; Product No. 547999) having a glass transition temperature of 123 °C and melt flow index of 2.2 g/10 min. Titanium dioxide (in puriss grade having a melting temperature of >350 °C and an average particle size of ~150 nm) used in this study has also been supplied by Sigma Aldrich (Product No. 14207).

### 2.2. Experimental Method and Design

#### 2.2.1. Method

##### Injection Molding Technique

HDPE nanocomposites enriched with variable concentrations of nano-TiO_2_ were prepared. Initially, under the influence of high shear, HDPE and TiO_2_ nanoparticles were blended together in order to provide a homogenous mixture of the constituents of the nanocomposites. The resulting dry mixture was then transferred into the injection molding machine (Ray-ran Test Sample Injection Molding Press, Warwickshire, UK) through a nozzle inside the barrel, which was maintained at a specified barrel temperature. Maintaining its specified period of residence inside the barrel, the melted polymer blend was then poured into the dumbbell-shaped specimens using a semi-automatic plunger.

##### Mechanical Testing

Injection molded dumbbell-shaped specimens of a gauge length of 20 mm were analyzed in a universal testing machine by MTS. Analysis was carried out at room temperature with a cross head speed of 5 mm/min and a load cell of 100 kN; tensile tests were carried out up to fracture.

##### Surface Characterization

**Figure 1:**
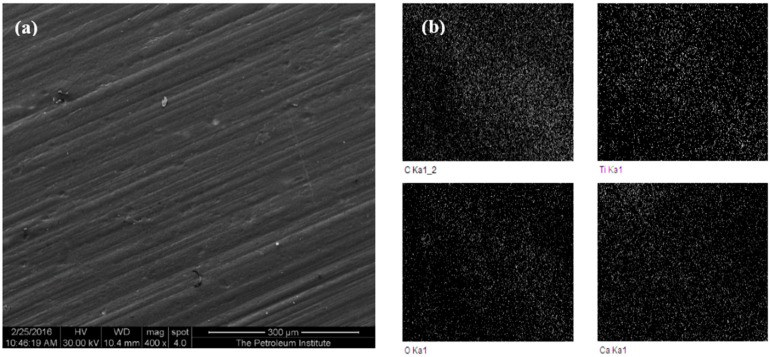
(**a**) Secondary electron SEM micrograph of HDPE composites with 5 wt % TiO_2_ prepared at a barrel temperature of 250 °C with a residence time of 50 min; (**b**) elemental mapping by EDS showing the constituent elements of the nanocomposites (the percentage of TiO_2_ was found to be at 80.88%). (Scale bar in (b)).

The prepared specimens were analyzed under a scanning electron microscope (SEM) equipped with an energy dispersive spectroscope (EDS) to confirm the presence of titanium dioxide and its even distribution into the polymeric matrix. Figure 1 shows the SEM and EDS mapping of a representative HDPE nanocomposite with 5 wt % titania filler.

#### 2.2.2. Experimental Design

The effect of the different control variables of injection molding in this design were quantified by measuring the three response variables, including yield strength (*σ*_y_, MPa), Young’s modulus (E, MPa) and elongation (% *δ*); these response variables were chosen to assess the injection molding performance based on their significance in biomedical applications. The key injection molding parameters under this study are the concentration of TiO_2_ (A, %), barrel temperature (B, °C), residence time (C, min) and holding time (D, s), where each of the factors was examined under three different levels. Khan et al. [16] studied the optimal injection molding parameters by studying virgin HDPE under the influence of varying melt temperature, holding pressure, injection time and holding time [16].

As mentioned earlier, the Taguchi method (orthogonal arrays) is a very useful technique in designing the layout of experiments based on the control factor levels and the degree of freedom [16]. The degree of freedom for the four-factor design at three levels was found to be 8, by ignoring the interactions of the parameters amongst each other. Subsequently, an L_9_ array was considered to design the experiments based on nine different runs. Table 1 represents the four controlled factors along with the three levels, and Table 2 shows the nine runs along with the measured responses.

#### 2.2.3. Grey Relational Analysis

**Figure 2:**
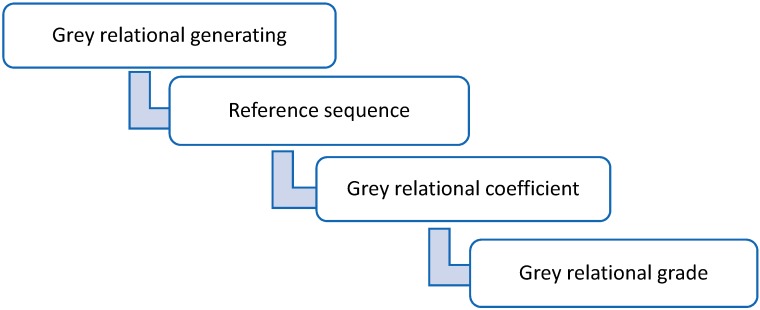
Procedure of the grey relational analysis [38].

Grey relational analysis, as described earlier, is an optimization tool for a multiple characteristic system, whereby it processes the data into a comparability sequence that allows the grey relational coefficient to be calculated when all attributes are compared to the reference or the ideal target sequence [38]. The coefficients are finally translated to a unified grey relational grade, the highest value of which determines a particular alternative or experiment to be the best. Figure 2 outlines the procedures involved in grey relational analysis [16].

The data pertaining to any particular process require some level of pre-processing before they can be translated into grey relational terms; this process is often known as grey relational generation [16,38]. In a multiple attribute problem, if a particular process is represented by m experiments and n attributes, then the original reference sequence and the sequence for comparison can be denoted by $x_{o}\left(k\right)\;\text{and}\;x_{i}\left(k\right)\;\text{for}\;i=1,2,\ldots ,\;m;k=1,2,\ldots ,\;n,\text{ respectively}.$ This data pre-processing converts the original sequences into a comparable sequence that is normalized between the values of between zero and one [16,32]. The following equations are used to carry out the normalization according to the nature of the particular attribute:

(1) $${x_{i}}^{*}\left(k\right)=\;\frac{\;{x_{i}}^{0}\left(k\right)-min\;{x_{i}}^{0}\left(k\right)}{max\;{x_{i}}^{0}\left(k\right)-\;min\;{x_{i}}^{0}\left(k\right)}\;$$

Equation (1) is used for the-larger-the-better attributes.

(2) $${x_{i}}^{*}\left(k\right)=\;\frac{max\;{x_{i}}^{0}\left(k\right)-{x_{i}}^{0}\left(k\right)}{max\;{x_{i}}^{0}\left(k\right)-\;min\;{x_{i}}^{0}\left(k\right)}\;$$

Equation (2) is used for the-smaller-the-better attributes.

(3) $${x_{i}}^{*}\left(k\right)=\;1-\frac{\left|\;{x_{i}}^{0}\left(k\right)-{x_{i}}^{0}\right|}{max\;{x_{i}}^{0}\left(k\right)-{x_{i}}^{0}}\;$$

Equation (3) is used for the-closer-to-the-desired-value, where, ${x_{i}}^{*}\left(k\right)$ is the value of the particular attribute after grey relational generation (data pre-processing), $max\;{x_{i}}^{0}\left(k\right)$ and $min\;{x_{i}}^{0}\left(k\right)$ are the maximum and minimum value of the $\;{x_{i}}^{0}\left(k\right)$ range, respectively, and $\;{x_{i}}^{0}$ is the desired value [32].

## 3. Analysis and Discussion of the Experimental Results

### 3.1. Optimal Parameters for Injection Molding Composites

The experimental results for the measured values of the target factors (obtained through uniaxial tensile testing) are presented in Table 3. As mentioned before, each run, treated as a comparability sequence, was processed according to the grey relational generating procedure by representing the responses with certain attributes. Generally, yield strength (*σ*_y_, MPa), Young’s modulus (E, MPa) and elongation (% *δ*) are considered to be positive variables for the design of bone implants and, therefore, were attributed “larger-the-better” for data pre-processing using Equation (1); the processed parameters are shown in Table 4.

The calculation of the grey relational coefficient is a crucial step before proceeding to the final step of determining the grey relational grade. The coefficient for each sequence takes into account the difference between each of the comparability entries for a particular experimental run. It determines how close ${x_{i}}^{*}\left(k\right)$ is to ${x_{0}}^{*}\left(k\right)$, and the larger the value of γ, the closer these two variables are. Moreover, the distinguishing coefficient denoted by ξ serves the purpose of expanding and compressing the range of the grey relational coefficients; it is important to note that regardless of the choice of ξ, the ranking order of γ for a particular series remains the same. In the case of the equivalent weighting of process parameters, the distinguishing coefficient is commonly taken as 0.5 [38]. The coefficients and the respective deviation sequences were calculated using Equation (4), and the results are presented in Table 5.

(4) $$\gamma \;\left({x_{0}}^{*}\left(k\right),{x_{i}}^{*}\left(k\right)\right)=\frac{{∆}_{min}+\xi {∆}_{max}}{{∆}_{0i}\left(k\right)+\xi {∆}_{max}}$$

$$0\;<\;\gamma \;\left({x_{0}}^{*}\left(k\right),{x_{i}}^{*}\left(k\right)\right)\;\leq 1$$

where,

$${∆}_{0i}\left(k\right)=\left|{x_{0}}^{*}\left(k\right)-{x_{i}}^{*}\left(k\right)\right|$$

$${∆}_{max}=\left|{x_{0}}^{*}\left(k\right)-{x_{j}}^{*}\left(k\right)\right|$$

$${∆}_{min}=\left|{x_{0}}^{*}\left(k\right)-{x_{j}}^{*}\left(k\right)\right|$$

ξ is the distinguishing coefficient ∈[0,1].

Finally, the grey relational grade is an indicator of the degree of similarity between the comparability sequence and the reference sequences of each factor level [25]. Hence, if a comparability sequence for an alternative gets the highest grey relational grade with the reference sequence, this means that the comparability sequence is most similar to the reference sequence, and that alternative would be the best alternative or the optimum combination of a set of parameters. It is also known as the weighted sum of the coefficients and is calculated using Equation (5), and the results are shown in Table 6.

(5) $$\Gamma \left({x_{0}}^{*},{x_{i}}^{*}\right)=\overset{n}{\underset{k=1}{\sum }}\beta \gamma \left({x_{0}}^{*}\left(k\right),{x_{i}}^{*}\left(k\right)\right)\;$$

A response table of the Taguchi method was employed to calculate the average grey relational grade for each injection molding parameter level. This was done by selecting the grey relational grades corresponding to the different factor levels in each column and taking an average of the grades at the same level. For example, Parameter A was set at Level 1 in the first, second and third experimental runs of the first column in the orthogonal array, as seen in Table 6, and therefore, the calculation is as follows:

$$\bar{A_{1}}=\frac{0.5094+0.4832+0.4688}{3}=0.4871$$

Using the same method, the averages were calculated for all injection molding control variables at the specified levels; these are presented in Table 7. As mentioned above, the grades reveal the level of correlation between the reference and comparability sequences, and a higher value means that there is a strong correlation. Based on this, it is possible to select a combination of optimum operating parameters that result in the largest overall response. According to Table 7, A_2_, B_2_, C_1_ and D_3_ indicate the highest grey relational grade for the control factors A, B, C and D, respectively. To conclude, 5 wt % of titanium dioxide, a barrel temperature of 225 °C, a residence time of 30 min and a holding time of 20 s are the optimal processing parameters for injection molded nanocomposites.

In a similar study, Khan et al. [16] studied the feasibility of using recycled HDPE as an alternative to using the virgin form of the polymer by optimization using grey relational analysis based on the Taguchi L_9_ experimental design; the performance variables for optimization were chosen to be the tensile, compressive and flexural strength. The analyses concluded that a melt temperature of 240 °C and a holding time of 30 s were the optimum conditions for the injection molding process. Moreover, Yang [24] optimized the injection molding process for the mechanical properties of polycarbonate composites reinforced with short glass fibers and PTFE. Based on the L_9_ Taguchi method, it was concluded that a melt temperature of 270 °C and a filling time of 3 s were found to be the optimum. Furthermore, Lin et al. [23] analyzed the effect of different injection molding parameters on the wear and tensile properties of polypropylene composites and via the Taguchi and design of experiments (DOE) method, concluding that 210 °C was the optimum melt temperature. Ozcelik et al. [53] studied the influence of injection molding parameters on ABS (acrylonitrile-butadiene-styrene) molds using S/N ratios based on Taguchi and found that the melt temperature had the most significant effect on the modulus, tensile strength, yield strength and the tensile strain at break of the molds.

### 3.2. Analysis of Variance

To investigate the significance of the injection molding operating conditions on the multiple performance characteristics, the analysis of variance (ANOVA) was conducted for the average grey relational grade responses at the 95% confidence level. The sum of squares (SS) and mean square (MS) were calculated along with the F-value (Fisher’s), and the results are presented in Table 8. According to Table 8, it can be noticed that the highest contribution is associated with Factor A, and it can be deduced that the concentration of titanium dioxide nanoparticle filler has the most significant effect on the mechanical properties of injection molded HDPE/TiO_2_ nanocomposites when all other control factors are considered simultaneously.

After the selection of the optimum processing factors of injection molding, the final step involves the confirmation experiments that verify the optimal combination of TiO_2_ concentration, barrel temperature, residence time and holding time. From the grey relational analysis, the conditions A_2_B_2_C_1_D_3_ were found to be the best combination; and these process conditions were used to fabricate the HDPE/TiO_2_ nanocomposites, which were subjected to tensile tests. According to the tensile testing results, the three response variables, such as yield strength, modulus of elasticity and elongation, were 23.75 MPa, 512.628 MPa and 684.1%, respectively. In summary, it can be inferred that the combination produced improved results when compared to the initial experimental runs.

## 4. Conclusions

The primary objective of this study was to optimize the injection molding process conditions for the fabrication of HDPE/TiO_2_ nanocomposites for biomedical applications. The concentration of TiO_2_ nanofiller, barrel temperature, residence time and holding time were chosen as the control parameters, while mechanical properties, such as yield strength, modulus of elasticity and elongation, were selected to be the representative performance characteristics. Based on the L_9_ Taguchi orthogonal array design of experiments and performing grey relational analysis, a TiO_2_ concentration of 5 wt %, a barrel temperature of 225 °C, a residence time of 30 min and a holding time of 20 s were found to be the optimum operating variables. Furthermore, ANOVA results showed that the percentage of nano-TiO_2_ filler was the most significant control factor in the injection molding of HDPE/TiO_2_ nanocomposites.

## Figures and Tables

**Table 1:** Experimental factors and factor levels.

Factor Levels	Control Factors			
	Concentration of Nano-TiO_2_ (A, %)	Barrel Temperature (B, °C)	Residence Time (C, min)	Holding Time (D, s)
1	1	200	30	16
2	5	225	50	18
3	10	250	70	20

**Table 2:** Taguchi orthogonal L_9_ array.

Run No.	Concentration of Nano-TiO_2_ (A, %)	Barrel Temperature (B, °C)	Residence Time (C, min)	Holding Time (D, s)
**1**	1	1	1	1
**2**	1	2	2	2
**3**	1	3	3	3
**4**	2	1	2	3
**5**	2	2	3	1
**6**	2	3	1	2
**7**	3	1	3	2
**8**	3	2	1	3
**9**	3	3	2	1

**Table 3:** Mechanical testing results.

Run No.	Yield Strength (*σ*_y_, MPa)	Young’s Modulus (E, MPa)	Elongation (% *δ*)
**1**	20.9 ± 0.2	384.3 ± 16.4	576.9 ± 31.7
**2**	20.5 ± 0.5	364.6 ± 13.4	580.8 ± 29.2
**3**	19.3 ± 0.7	363.2 ± 19.9	598.6 ± 41.4
**4**	22.1 ± 0.6	481.8 ± 16.6	492.5 ± 30.0
**5**	22.6 ± 0.3	346 ± 23.6	646.8 ± 36.6
**6**	21.3 ± 0.6	401.1 ± 12.6	622 ± 35.8
**7**	20.8 ± 0.6	438.5 ± 20.6	412 ± 35.0
**8**	21.5 ± 0.7	425.9 ± 17.0	628.6 ± 42.0
**9**	19.9 ± 0.2	422.9 ± 13.5	559.3 ± 31.4

**Table 4:** Comparability sequence after data pre-processing.

Experimental Run	Orthogonal Array				Comparability Sequence		
	A	B	C	D	Yield Strength (*σ*_y_, MPa)	Young’s Modulus (E, MPa)	Elongation (% *δ*)
**1**	1	1	1	1	0.4815	0.2820	0.7023
**2**	1	2	2	2	0.3704	0.1372	0.7189
**3**	1	3	3	3	0.0000	0.1268	0.7947
**4**	2	1	2	3	0.8344	1.0000	0.3428
**5**	2	2	3	1	1.0000	0.0000	1.0000
**6**	2	3	1	2	0.5926	0.4058	0.8944
**7**	3	1	3	2	0.4444	0.6813	0.0000
**8**	3	2	1	3	0.6667	0.5885	0.9225
**9**	3	3	2	1	0.1852	0.5663	0.6273

**Table 5:** Reference sequences and calculated grey relational coefficients.

Experimental Run	Reference Sequence			Grey Coefficients		
	Yield Strength (*σ*_y_, MPa)	Young’s Modulus (E, MPa)	Elongation (% *δ*)	Yield Strength (*σ*_y_, MPa)	Young’s Modulus (E, MPa)	Elongation (% *δ*)
**1**	0.5185	0.7180	0.2977	0.4909	0.4105	0.6268
**2**	0.6296	0.8628	0.2811	0.4426	0.3669	0.6401
**3**	1.0000	0.8732	0.2053	0.3333	0.3641	0.7089
**4**	0.1656	0.0000	0.6572	0.7512	1.0000	0.4321
**5**	0.0000	1.0000	0.0000	1.0000	0.3333	1.0000
**6**	0.4074	0.5942	0.1056	0.5510	0.4569	0.8256
**7**	0.5556	0.3187	1.0000	0.4737	0.6107	0.3333
**8**	0.3333	0.4115	0.0775	0.6000	0.5486	0.8658
**9**	0.8148	0.4337	0.3727	0.3803	0.5355	0.5730

**Table 6:** Calculated grey relational grades of all of the experimental runs.

Experimental Run	Orthogonal Array				Grey Relational Grades
	A	B	C	D	
**1**	1	1	1	1	0.5094
**2**	1	2	2	2	0.4832
**3**	1	3	3	3	0.4688
**4**	2	1	2	3	0.7278
**5**	2	2	3	1	0.7778
**6**	2	3	1	2	0.6112
**7**	3	1	3	2	0.4726
**8**	3	2	1	3	0.6715
**9**	3	3	2	1	0.4963

**Table 7:** Response table of the grey relational grades for the target variables.

Control Levels	Response Factors			
	A	B	C	D
**1**	0.4871	0.5699	0.5973	0.5945
**2**	0.7056	0.6441	0.5691	0.5223
**3**	0.5468	0.5254	0.5730	0.6227

**Table 8:** ANOVA results.

Control Factors	Level 1	Level 2	Level 3	Degrees of Freedom	Sum of Squares	Mean Square	F	Percentage Contribution (%)
A	0.4871	0.7056	0.5468	2	0.0765	0.0383	0.0383	66.20
B	0.5699	0.6441	0.5254	2	0.0216	0.0108	0.0108	18.67
C	0.5973	0.5691	0.573	2	0.0014	0.0007	0.0007	1.21
D	0.5945	0.5223	0.6227	2	0.0161	0.0080	0.0080	13.92
Error				0	0.0000			
Total				8	0.1156	0.0578	0.0578	100.00
